# Ultrasound-Guided Percutaneous Ethanol Injection Protocol to Treat Solid and Mixed Thyroid Nodules

**DOI:** 10.3389/fendo.2016.00052

**Published:** 2016-06-06

**Authors:** João Soares Felício, Antônio Maria Silva Conceição, Flávia Marques Santos, Michelle Masuyo Minami Sato, Fabíola de Arruda Bastos, Ana Carolina Contente Braga de Souza, Camila Cavalcante Koury, João Felício Abrahão Neto, Franciane Trindade Cunha de Melo, Carolina Tavares Carvalho, Thaís Pontes Arbage, Antonio Bentes de Figueiredo Junior, Hana Andrade de Rider Brito, Marcelo Oliveira Mourão Júnior, Fabricio de Souza Resende, Amanda Soares Peixoto, Karem Miléo Felício

**Affiliations:** ^1^Endocrinology Division, University Hospital João de Barros Barreto, Federal University of Pará, Belém, Brazil

**Keywords:** thyroid nodules, percutaneous ethanol injection, alcoholization, US-guided, nodule volume reduction

## Abstract

**Context:**

Ultrasound (US)-guided percutaneous ethanol injection (PEI) has been proposed for treatment of benign thyroid nodules (TNs). However, there is no consensus for the optimal amount of ethanol injection, number of applications, and time to re-evaluation in order to achieve maximum volume reduction with minimum adverse effects.

**Objective:**

The purpose of the present study was to analyze the effectiveness of an US-guided PEI protocol to treat solid and mixed TNs based on a new target outcome.

**Patients and methods:**

We performed a prospective study evaluating the results of PEI in 52 patients with benign solid and mixed TNs. The ethanol dose was fixed in 30% of the nodular volume per session. Patients returned 1 month after each session for US re-evaluation. Therapeutic success was defined as volume reduction of at least 30% associated with disappearance of clinical symptoms and a complete esthetic satisfaction reported by the patient.

**Results:**

We performed a mean of 2.8 ± 1.9 PEI sessions, with an average total volume of ethanol injected of 9.1 ± 10.3 ml, and a follow-up time of 10.0 ± 8.7 months. There was a reduction of at least 50% of the initial nodular volume in 33 patients (63.5%). In 11 patients (21.2%), the reduction did not reach 50% (mean reduction of 31 ± 11%), but 6 of them reported esthetically satisfactory results and treatment was stopped. Our therapeutic success rate considering the patients with esthetic improvement was 75%. There were no severe complications.

**Conclusion:**

Our protocol is effective and safe to treat solid and mixed benign TNs based on our established outcome.

## Introduction

Thyroid nodules (TNs) are common entities, often detected in clinical practice, with a largely variable prevalence, depending on the diagnostic method (1, 2). Epidemiological studies have shown that 4–7% of women and 1% of men living in iodine-sufficient regions present palpable TNs (3, 4), whereas ultrasound (US) detects nodules in up to 76% of the adult population, with a greater incidence in women and elderly people (5–9).

Most TNs are benign (94–96%) (10, 11) small and remain asymptomatic, being usually managed only by observation and follow-up. A proportion of them, however, may lead to local compressive symptoms or esthetic complaints, due to significant enlargement, requiring specific intervention (1, 12). The best treatment for benign nodules remains uncertain (13). Alternatively to thyroidectomy, the use of non-surgical, image-guided, minimally invasive methods has been proposed (12–14), such as US-guided percutaneous ethanol injection (PEI) (15, 16).

This method has been suggested to be an effective, safe, and inexpensive outpatient procedure for the treatment of benign TNs, leading to reduction of nodular volume, with a low rate of early and late clinical complications (3, 17–29). It has been recommended as the first-line therapy of symptomatic and recurrent thyroid cysts with benign cytology (30), and may also be used in solid nodules, particularly in case of contraindication to or refusal of surgery and radioiodine therapy (2, 31–34). However, a standard US-guided PEI protocol is yet to be established, since there is no consensus definition for the optimal amount of ethanol to be injected, the number of applications, and the intervals between sessions in order to achieve maximum volume reduction with minimum adverse effects.

Most articles in literature have considered a nodule volume reduction of at least 50% as a partial success and more than 90% as a complete outcome (34). It is well known that an established benign thyroid nodule rarely becomes too malignant during the lifelong (35). Therefore, it makes more sense to evaluate the treatment success of this pathology based on disappearance of clinical symptoms and a complete esthetic satisfaction to stop PEI applications, independently of volume percent reduction. Our protocol was based mainly on this idea.

Therefore, the purpose of the present study was to analyze the effectiveness of an US-guided PEI protocol to treat solid and mixed TNs based on that concept of treatment outcome.

## Materials and Methods

### Study Design and Patients

We performed a prospective study evaluating the results of an original US-guided PEI protocol in patients with solid and mixed TNs at Endocrinology Department of Federal University of Pará, from January 2011 to January 2016. We included 52 patients diagnosed with TNs and normal thyroid function, confirmed to be benign by fine-needle aspiration biopsy (FNAB). Mixed TNs were only included when the solid component was >50% of the nodular volume. All patients presented compressive local symptoms and/or esthetic complaints. Exclusion criteria were the presence of malignancy or suspected malignancy detected by FNAB. Cystics and autonomous TNs were not included. The study was approved by the local ethics committee-University Hospital João de Barros Barreto, Federal University of Pará.

### Ultrasound-Guided Percutaneous Ethanol Injection

Patients were placed in a supine position with mild neck extension. After skin sterilization, a 22 gage needle (Becton Dickinson 30 × 7) was inserted through the epidermis to the thyroid pericapsular space under US guidance, reaching the center of the target lesion. Then, 99% ethanol was slowly instilled into the nodule, respecting the patient’s tolerance. Ethanol diffusion through the lesion was monitored as intense echogenicity in real-time observation by US. The procedure was stopped if ethanol leaked out of the nodule or if the patient complained of severe pain. None of 52 patients were dropped out through this procedure, and no cases of severe pain were reported. The procedure was carried out without local anesthesia at the puncture site.

The amount of ethanol injected was equivalent to approximately 30% of the nodule volume, estimated by ultrasonography before each application. The nodular volume (milliliter) was obtained by multiplying the product of the longitudinal, lateral, and anteroposterior axes (centimeter) by the constant 0.52.

In mixed nodules, we removed the liquid portion guided for the US, and the nodular volume was calculated. In cases of septated nodules, the greatest possible liquid part was removed. If patients had a multinodular goiter, treatment was only performed in the dominant nodule. In all alcoholization sessions, the amount of injected ethanol did not exceed 5 ml.

Nodular volume reduction was calculated according to the following formula: [Pretreatment volume (milliliter) − Post-treatment volume (milliliter)] × 100%/Pretreatment volume (milliliter). The PEI sessions were followed by monthly assessments of nodular volume by US, and clinical evaluation.

Therapeutic success was defined as volume reduction of at least 30% associated with disappearance of clinical symptoms and a complete esthetic satisfaction reported by the patient. The procedure was considered ineffective when the nodule did not show significant volume reduction (30%) and the patient did not refer esthetic improvement after at least three sessions of PEI and a follow-up period of at least 6 months.

The new information of our study is a protocol that used ethanol fixed doses, but with shorter periods of re-evaluation by US and consequently more frequent applications based not just in nodule volume. We have also taking in consideration the disappearance of clinical symptoms and a complete esthetic satisfaction to stop PEI applications.

### Statistical Analysis

Categorical variables were presented as frequency (percentage). All normally distributed values were given as mean ± SD and all other values were given as median (range). The Wilcoxon test was applied to compare thyroid nodules initial and final volumes. A two-sided *p* < 0.05 was considered statistically significant. Data were analyzed using Sigma Stat version 3.5 (Jandel Scientific Corporation, Chicago, IL, USA) and Statistical Package for the Social Sciences version 21.0 (IBM, Chicago, IL, USA).

## Results

Our study group consisted of 51 women (98%) and 1 man, with a mean age of 45.2 ± 16.6 years. Among TNs, 19 (36, 5%) were mixed and 33 (63, 5%) solid.

We performed a mean of 2.8 ± 1.9 PEI sessions, with an average total volume of ethanol injected of 9.1 ± 10.3 ml, and a follow-up time of 10.0 ± 8.7 months. Data regarding the US evaluation of the TNs before and after treatment are described in Table 1. There was a reduction of at least 50% of the initial nodular volume in 33 patients (63, 5%). In 11 patients (21, 2%), the reduction did not reach 50% (mean reduction of 31 ± 11%). In eight patients (15, 3%), the nodular volume either increased or remained unchanged.

**Table 1:** **US evaluation of the thyroid nodules before and after treatment (*n* = 52)**.

**Variables**	**Mean ± SD**
Initial volume (cm^3^)*	13.2 ± 12.5
Final volume (cm^3^)	7.1 ± 9.9
Volume reduction (cm^3^)	6.2 ± 8.2
Volume reduction (%)	44.5 ± 47.4
Number of US evaluations	5.0 ± 2.7

*p < 0.01 vs. final volume.

Additionally, 6 of the 11 patients who did not achieve 50% of nodular volume reduction reported esthetically satisfactory results with a reduction of 30–50%, continuing in the protocol with only clinical follow-up. Considering all patients with nodular volume reduction ≥50% (*n* = 33) and those who reported esthetic improvement with reduction of 30–50% (*n* = 6), the therapeutic success rate of our PEI protocol increases to 75% (39/52). We compared our results with other series using PEI for treating TNs (Table 2).

**Table 2:** **Outcomes of US-guided PEI for treatment thyroid nodules**.

**Reference**	**Country**	**Number of nodules**	**Type of nodule**	**Volume of ethanol injected (mean ± SD)**	**Number of sessions (mean ± SD)**	**Therapy success (%)**	**Follow-up (months)**
This research	Brazil	52	Mixed and solid	9.1 ± 10.3 ml (total)	2.8 ± 1.9	75	10 months
Perez et al. (2)	Brazil	120	Cystic, mixed, solid, and autonomous	6.2 ml (total)	2.8	70	6 months–11 years
Basu et al. (36)	India	60	Cystic and mixed	NA	NA	78	12.3 months
Kim et al. (33)	South Korea	30	Solid	2.3 ml (first session)	1.2	60	15 months
Tarantino et al. (32)	Italy	124	Cystic, mixed, and solid	14 ml (total)	4	66	60 months
Del Prete et al. (37)	Italy	98	Cystic	NA	NA	94	9 years
Cho et al. (38)	South Korea	22	Cystic and mixed	NA	1.2	68	3.5 months
Zingrillo et al. (39)	Italy	43	Cystic	NA	NA	93	5 years
Yasuda et al. (40)	Japan	61	Cystic and mixed	NA	1.26	72.1	1–6 months
Bianchini et al. (41)	Brazil	26	Solid	10 ml	5	74	12 months
Lee et al. (34)	South Korea	198	Solid	NA	2.2 ± 1.4	89	36.2 months

*NA, not available*.

Complications associated with PEI were reported in two patients, and included one hematoma with full recovery during the follow-up period. Some patients complained of local pain at the injection site. However, pain was mild and transient, and there were no severe complications, such as massive hemorrhage, vocal cord paralysis, or transient thyrotoxicosis.

## Discussion

In the present study, we realized an US-guided PEI protocol for the treatment of benign mixed and solid TNs, with a 75% success rate in a short follow-up period. The procedure was safety in comparison to other methodologies previously described.

The use of USG-guided PEI as an alternative to surgery in the management of benign thyroid nodules was first proposed in 1990, by Livraghi et al. (42). Ethanol acts as a primary sclerosing agent (43) promoting cellular dehydration, coagulation necrosis and small vessel thrombosis, which is followed by progressive fibrosis and nodular volume reduction (18, 44, 45).

This procedure is well established as the first-line option for treatment of benign thyroid cysts that recur after evacuation (30), and has also been used in solid nodules, particularly in patients who are not candidates for surgery or radioiodine therapy (2, 32–34, 43).

Perez et al. (2), using PEI, have found a greater mean volume reduction in cystic (66.7%) compared to mixed (63%) and solid TNs (52.9%). The solid nodules (*n* = 37) were less likely to completely respond compared to the others nodules. They have found a global satisfactory response in 70% of cases. Nevertheless, it was not reported the success rate specifically in solid TNs. Furthermore, Lee et al. (34), in a large study with 198 patients, demonstrated a relevant reduction in solid TNs in 89% of cases. Even though they needed a long follow-up period of 3 years and some patients were submitted to six applications, it suggests that ethanol ablation still could be a good option to treat solid TNs since we achieve a standard protocol. Finally, Bianchini et al., in 2003, treated 26 solid TNs with ethanol injection amount according to TNs volume (41). In our protocol, we also used ethanol fixed doses, but with shorter periods of re-evaluation and consequently more frequent applications. These differences contributed to satisfactory success rates, with a larger number of patients in a shorter follow-up period.

When compared to other methods of treatment, PEI has shown more effective results and fewer complications. Four controlled studies demonstrated a 75–85% success rate after an average of two PEI sessions, compared with a 7–38% success rate in controls treated by simple cyst evacuation or saline injection (46–49). Additionally, a 12-month randomized study evidenced that the use of levothyroxine for thyroid stimulating hormone (TSH) suppression had no significant effect on nodule reduction, while a single PEI session occasionally induces a nearly 50% volume reduction (50). Furthermore, Zingrillo et al. (51) demonstrated a greater nodule volume reduction percentage in patients treated with PEI in comparison to radioiodine therapy, which, in addition, is accompanied by a 10–40% risk of hypothyroidism (51–53). Other therapies have been reported as a good alternative to treat solid TNs. Particularly, percutaneous laser ablation and radiofrequency have showed good results (54–56). Although several studies have demonstrated significant volume reduction of thyroid nodules treated with PEI, the protocols proposed are a lot heterogeneous in concern to nodules size and type, doses of ethanol used, number of injections, intervals between sessions, and follow-up length (50, 57, 58).

The highly variable success rate reported in different series might be related to the heterogeneous nature of thyroid nodules evaluated, ranging from purely cystic to purely solid nodules (36). The best results have been obtained in the treatment of large or symptomatic cystic nodules. In a prospective study with a mean follow-up of 5 years, volume reductions of at least 50% were achieved in 40/43 (93%) of patients with cystic nodules treated by PEI (39). Del Prete et al. (37) found similar results, with volume reduction >50% induced by PEI in 92/98 (94%) patients with symptomatic thyroid cysts. Outcomes have been reported to be worst in solid TNs, which is likely to be due to poor diffusion of ethanol in the solid tissue along with early wash out of ethanol due to the increased vascularity of a solid nodule as compared to a thyroid cyst (59). Our series included patients mixed and solid TNs, which might explain the lower success rate of PEI treatment when compared to other authors that assessed only simple or complex cysts. Additionally, the amount of ethanol to be injected has not been well established, with great variability between each protocol (30–100% of the initial nodular volume), which might also be related to the different outcomes observed with PEI treatment (2, 33, 38, 46, 47, 60–62). In the present study, the ethanol dose calculated was 30% of the nodular volume measured before the beginning of each session. Some authors have demonstrated a positive correlation between the volume of ethanol instilled and the volume reduction rate of cysts, without an increase of side effects with more ethanol injected (38, 59). However, most authors do not recommend administering a single dose of more than 10 ml of ethanol (63). In our study, we basically adopted a short time between evaluations. Therefore, the patients were submitted to a higher frequency of applications according to evaluation results. It may have contributed to our success rate in a short follow-up period compared to other studies.

Percutaneous ethanol injection is a safe procedure without serious complications (2). The most common reported adverse effects, caused by ethanol leakage into the surrounding tissues (63), are local pain, dysphonia, flushing, and dizziness (46, 47, 49). Although rare, other complications have been reported, such as recurrent nerve palsy, Graves’ disease, Graves’ orbitopathy, Horner’s syndrome, facial dysesthesia with increased tear flow, necrosis of the larynx and skin, and impairment of post-PEI surgery due to local fibrosis (57, 64). Accordingly, in our study there were no severe or long-standing complications.

In conclusion, our US-guided PEI protocol is safe and effective for mixed and solid thyroid nodules when compared with other methodologies described, with a 75% success rate, in a short follow-up period and no major complications based on that new target idea. In addition, we need to increase the number of patients to confirm the effectiveness of this approach.

## Author Contributions

JF wrote, reviewed and edited the final version, and was responsible for submitting the manuscript. AS and KM are university professors and helped writing the paper. CK, JN, CC, TA, AJ, HB, MJ, FR, FS, MS, FB, FM, AP and AC who have contributed by creating the database and contacting patients. All authors read and approved the final manuscript, and agreed to its submission.

## Conflict of Interest Statement

The authors declare that the research was conducted in the absence of any commercial or financial relationships that could be construed as a potential conflict of interest.
